# In Vitro Propagation of *Isoëtes sabatina* (Isoetaceae): A Key Conservation Challenge for a Critically Endangered Quillwort

**DOI:** 10.3390/plants9070887

**Published:** 2020-07-14

**Authors:** Sara Magrini, Mattia M. Azzella, Rossano Bolpagni, Laura Zucconi

**Affiliations:** 1 Tuscia Germplasm Bank, Tuscia University, largo dell’Università—blocco C, 01100 Viterbo, Italy; zucconi@unitus.it; 2 National Agency for New Technologies, Energy and Sustainable Economic Development (ENEA)—Frascati Research Center, Via Enrico Fermi 45, 00044 Frascati, Italy; mattia.azzella@enea.it; 3 Department of Chemistry, Life Sciences and Environmental Sustainability, Parma University, Parco Area delle Scienze 11/a, 43124 Parma, Italy; rossano.bolpagni@unipr.it; 4 Department of Ecological and Biological Sciences, Tuscia University, largo dell’Università—blocco C, 01100 Viterbo, Italy

**Keywords:** aquatic lycophyte, freshwater plants, Mediterranean, reproduction, spore germination, sporeling production

## Abstract

*Isoëtes sabatina* is an aquatic quillwort endemic to Italy. It is one of the rarest quillworts in Europe, and is critically endangered due to restricted range and to the continuous decline of both population and habitat quality. This study aims to develop an optimized protocol to reproduce and grow *I. sabatina* sporelings. Mature and immature megaspores were mixed with mature microspores to evaluate the influence of the developmental stage on germination and sporeling development. Two substrates, distilled water and water-agar medium, were tested for germination and sporeling emergence, and three substrates, sand, lake sediment and water-agar, were tested for transplants. A high percentage of megaspore germination (a total of 79.1%) was obtained in both substrates, higher for mature than immature spores. A total of 351 sporelings were produced in distilled water and water-agar cultures, with similar percentages (64.5% and 69.6%, respectively). The development stage of the megaspores affected both germination and sporeling development. Sporeling emergence showed significantly higher percentages in mature megaspores than immature ones (69.6% vs. 11.6%, respectively), with 85% of germinated spores developing sporelings. Only transplants over water-agar medium were successful. This protocol could be useful for the propagation of sporelings as the key step towards the planning of in situ actions to save this Mediterranean quillwort from extinction.

## 1. Introduction

The family *Isoëteaceae* consists of a single genus, *Isoëtes* L., which currently includes about 250 species [1,2] occurring in lakes, wetlands, and terrestrial habitats all over the world, from the tropics to the subarctic [1,3]. Some species are widely distributed [4,5], while several others remain restricted to a few sites [6,7,8,9,10]. This genus also shows high local diversification and endemism rate [11]. 

Quillworts and their fragile habitats are known to be highly threatened worldwide and they can be key indicator species of aquatic ecosystem health [10,12,13]. An overall decline of *Isoëtes* populations has been reported for a large part of the extant species that are facing serious risks of extinction [6,8,10,14]. In particular, 50% of European *Isoëtes* have been assessed as threatened [13], and five out of the ten species occurring in Italy are endangered or critically endangered [15,16]. Climate change and environmental disturbances, such as eutrophication, pollution, habitat loss and alteration of hydrological regimes, are the primary threats to *Isoëtes* species worldwide [1,13,17,18]. 

**Figure 1:**
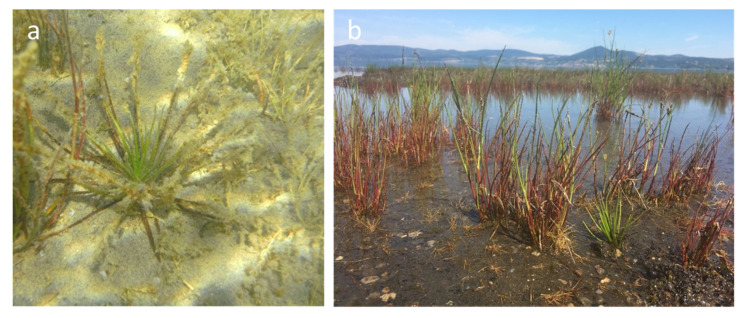
*Isoëtes sabatina* in Lake Bracciano (**a**) growing completely submerged on a sandy substrate at a depth of 1.5 m (summer 2013) and (**b**) completely emerged in the summer of 2017.

*Isoëtes sabatina* Troia and Azzella (Figure 1a) is a small aquatic quillwort endemic to Italy, occurring exclusively at the south-eastern coast of Lake Bracciano, a volcanic lake within the Regional Park of Bracciano and Martignano Lakes near the city of Rome [9]. Here, *I. sabatina* grows in an area of about 700 m^2^ on a sandy substrate at a water depth of 0.5–1.5 m, in a coenosis characterized by the structural dominance of *Juncus articulatus* L. and *Eleocharis acicularis* (L.) Roem. and Schult., and by the presence of *Baldellia ranunculoides* (L.) Parl. [9]. It is one of the rarest quillworts in Europe and it was assessed as critically endangered due to its restricted range and to the continuous decline of both its population and the quality of the habitat [9,13,15]. 

In 2017, all the Mediterranean countries faced an exceptional drought, which strongly affected Lake Bracciano (e.g., [19,20]). This event, concurrently with an excess of lake water extraction to meet the demands of the city of Rome, of which Lake Bracciano is a strategic water reserve, resulted in an extreme, and never previously recorded, reduction of the lake water level (up to ca. 2 m), which drastically affected the whole littoral zone and the survival of *I. sabatina* [21]. Consequently, a substantial decline of the *I. sabatina* population was recorded, provisionally evaluated at around 60% of the known individuals (Figure 1b), and, currently, no more than 400 plants survive in the wild. Moreover, as the climate forecasts predict an increase in warming and drying through the Mediterranean region (e.g., [22,23]), an increase in hydrological variability for Lake Bracciano is expected, reinforcing the risk of extinction of *I. sabatina* in the short term.

Such a state of emergence calls for urgent conservation actions, such as a reinforcement of the population of *I. sabatina* before the low number of individuals becomes critical and definitively compromises its survival in the wild. To counteract this critical situation, the Regional Park of Bracciano and Martignano Lakes and the Tuscia University immediately activated a program for in vitro conservation of this species at the Tuscia Germplasm Bank, which has long-term experience in conservation and reproduction of ferns (e.g., [24,25,26,27]), as the first step to recover *I. sabatina* from the brink of extinction. The in vitro plant propagation is well-established as a useful tool in conservation programs that include habitat restoration or reintroduction/reinforcement of plant populations [28]. It can be efficiently used to complement and support in situ methods, with major advantages over conventional propagation techniques as the rapid multiplication under controlled and pathogen-free conditions. Sometimes, it represents the only option for conserving certain highly endangered and rare species [29,30]. However, reports of successful sporeling production and growth have been described only for a few *Isoëtes* species, such as the Italian endemic *I. malinverniana* Ces. and De Not. [31], *I. lacustris* L. [32], and recently *I. cangae* J.B.S.Pereira, Salino and Stützel, endemic to the Brazilian Amazon [18], but none from the Mediterranean biogeographical region.

In this study, we investigated the reproductive biology and ex situ growth of *I. sabatina*. Here we provide (1) a method to reproduce a large number of sporelings from in vitro mixed spore culture (microspores and megaspores), (2) the description, quantification and timing of germination and sporeling emergence, (3) the evaluation of the germination and sporeling emergence of both mature and immature megaspores, and (4) the optimal transplanting method that enables the successful growth of the sporelings in the long term with a lower mortality rate. The ultimate goal was to develop an optimized protocol to reproduce and grow *I. sabatina* sporelings, providing a critical step towards population rescue and ex situ conservation, which can assist in the conservation efforts of this and other aquatic quillworts in Mediterranean areas.

## 2. Results

### 2.1. Spore Germination

**Figure 2:**
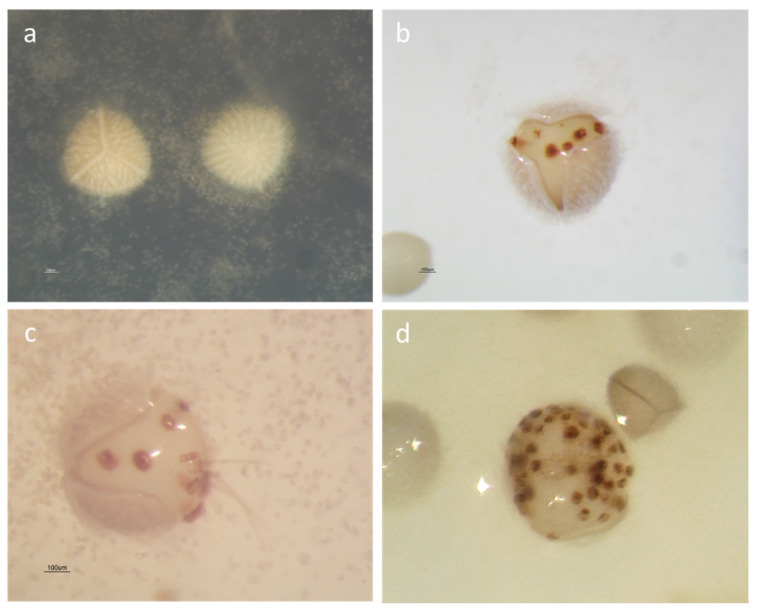
Spore germination of *Isoëtes sabatina*: (**a**) Initial adhesion between numerous microspores and megaspores; (**b**) endosporic germination of a megaspore split along the triradiate ridges, after two weeks of culture, with several archegonia (brown spots) developed on the exposed surface of the megagametophyte; (**c**) megagametophyte bearing some rhizoids, surrounded by multiple germinated microspores (empty), already split out to release spermatozoids; (**d**) non-fertilized megagametophyte with more than 50 archegonia 2 years and 10 months after sowing.

After 120 days of observations, no germination was observed in the cultures of spores from beached plants. All attempts failed because megaspores were too small and underdeveloped to germinate. On the other hand, both micro- and megaspores from mature plants germinated in the two substrates tested. Microspores germinated quickly after their release from the sporangia; thus, endosporic microgametophytes, ovoid in shape, developed within a few days, releasing spermatozoids in the water layer (Figure 2c). The megaspores showed great morphological changes during the culture. The spore wall was cracked by the expansion of the developing megagametophytes, reaching a 33–40% increase in equatorial diameter and split along the triradiate ridges (Figure 2b). Archegonia (visible as brown spots) developed on the exposed surface of the megagametophytes (Figure 2b) together with several rhizoids (Figure 2c). The first endosporic germination was observed in mature megaspores after 35 days of culture in water-agar medium, while immature megaspores started to germinate significantly later, after 69 days (Table 1). Continuous development of archegonia was observed in several non-fertilized megagametophytes showing 48–58 archegonia 2 years and 10 months after sowing (Figure 2d).

A total of 530 out of 670 megaspores germinated (79.1%) 120 days after sowing. Non-significant differences were detected between distilled water and water-agar cultures (Table 1). Similar germination percentages were recorded between the two substrates (G > 80%; Dunn’s test: *not significant, ns*) and only small differences were recorded in the time required for the first germination, with a smaller range in distilled water than in water-agar (T_G_: 40–43 vs. 35–60 days; Dunn’s test: *ns*).

Significant differences were recorded in water-agar cultures by comparing the germination indices according to the megaspore developmental stages (Table 1). G was significantly higher in mature relative to immature spores (82.1% vs. 65.6%, respectively; Dunn’s test: *p* < 0.05) and T_G_ was significantly lower (48.3 vs. 77.0 days, respectively; Dunn’s test: *p* < 0.05).

### 2.2. Sporeling Development

A total of 351 sporelings were produced in both distilled water and water-agar cultures, which is equal to 52.4% of all the sown megaspores and 66.2% of all the germinated ones. 

**Figure 3:**
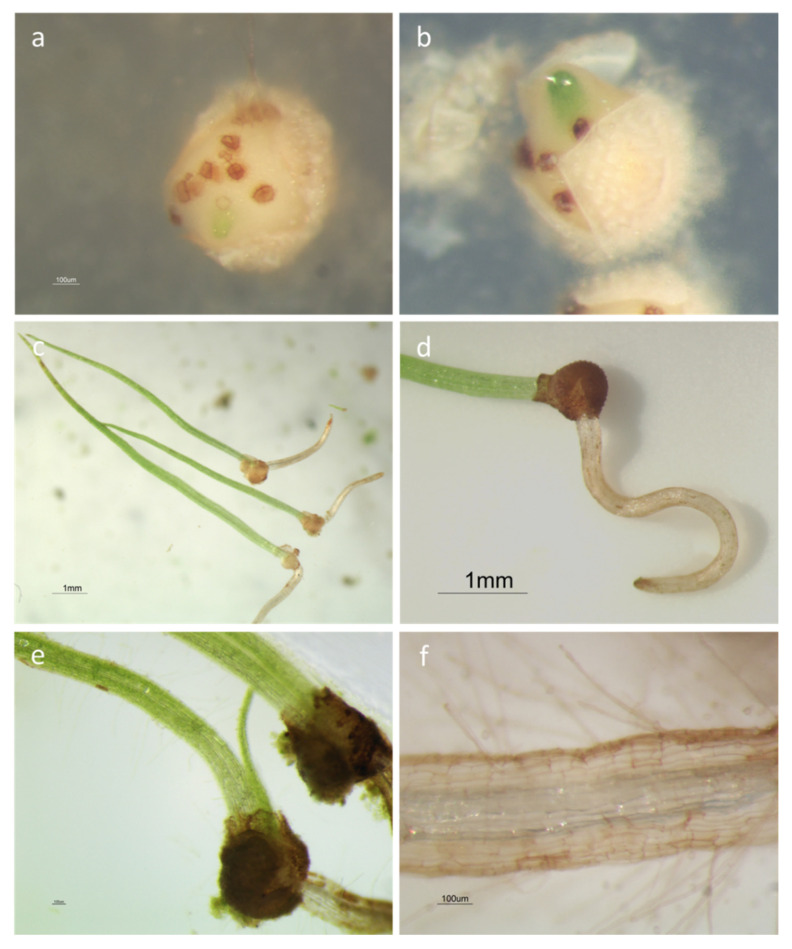
Sporophyte development from mixed spore cultures of *Isoëtes sabatina*: (**a**) the first stage of embryo development with the green apex of the primary leaf emerging from the megagametophyte; (**b**) the primary leaf protruding out the megagametophyte still enclosed within a large calyptra; (**c**) 20-day sporelings with the first leaves (7–10 mm) and roots (2–3 mm); (**d**) enlarged view, with the first leaf emerging from the calyptra and a root; (**e**) emergence of the second leaf from 35-day sporelings; (**f**) particular of a root bearing numerous root hairs; the central cavity is evident.

Sporeling emergence (Figure 3a,b) started earlier for mature spores than for immature ones (48 vs. 74 days after the sowing, respectively), 9–16 days after megaspore germination, independently from the spore developmental stage, and continuing for a further two months.

Leaves (Figure 3c) showed a sharp elongation during the first days (relative growth rate—RGR = 0.32 ± 0.02 mm/day), replaced by slower growth rates. The second leaf emerged approximately 10 days after the first one (Figure 3e). The root emergence (Figure 3d) was slower, emerging approximately 5–6 days later. Root hairs developed on the first root when it reached a length of at least 1–2 mm (Figure 3f).

#### 2.2.1. Effect of the Substrate on Sporeling Emergence

The use of two different substrates did not affect sporeling emergence. Lower emergence percentages (both SE (sporeling emergence) and SE_G_) and lower time for the observation of the first sporeling emergence (T_1_) were recorded in distilled water, with non-significant differences between the two substrates (Table 2; Dunn’s test: ns). A significantly higher percentage of sporelings per day (mean daily emergence—MDE) was recorded in distilled water cultures (Table 2; Tukey’s test: *p* < 0.001). 

#### 2.2.2. Effect of the Megaspores Developmental Stage on Sporeling Emergence

Highly significant differences were detected in eight out of ten indices used for the evaluation of the sporeling emergence from mature and immature megaspores on water-agar cultures. Data and results of the statistical analysis carried out on all the used indices are reported in Table 2. 

**Figure 4:**
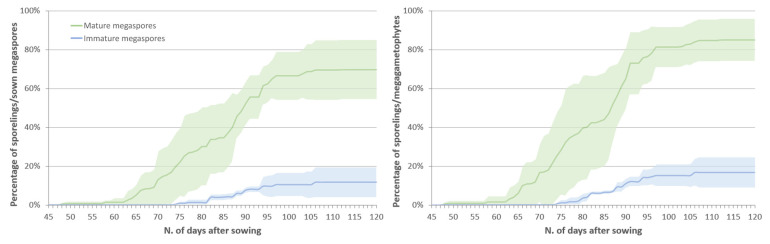
Time courses of the cumulative percentages of in vitro sporeling emergence from mature (green line) and immature (blue line) megaspores (**a**) and megagametophytes (**b**) of *Isoëtes sabatina* incubated in water-agar medium in a mixed spore culture. Data are represented as the average values with the shaded areas representing the standard deviation.

Approximately 50% of all the sown megaspores (mature and immature) and 64% of the megagametophytes successfully developed into sporelings. Sporeling emergence showed significantly higher percentages in mature megaspores than immature ones (SE = 70% vs. 12%, respectively; Dunn’s test: 0.001 < *p* < 0.01), with the percentage of sporeling emergence from germinated spores (SE_G_) reaching 85% in mature spores and only 17% in immature ones (Table 2, Figure 4; Dunn’s test: 0.001 < *p* < 0.01). Non-significant differences were detected in the mean emergence time (MET), which resulted as quite long (>80 days) for both mature and immature spores, and in the mean emergence rate (MER). The coefficient of variation of the emergence time (CVt) showed a low variability, with the calculated values being lower than 14%, and higher in mature than immature spores (Dunn’s test: *p* < 0.05). Other significant differences were detected in the velocity of the sporeling emergence, with a significantly lower time for the observation of the first sporeling (T_1_) from mature spores (59.5 vs. 85 days, respectively; Tukey’s test: *p* < 0.05) and with a significantly higher emergence index (EI; *t*-test: *p* < 0.05), mean daily emergence (MDE, Tukey’s test: *p* < 0.001) and peak value (PV, *t*-test: *p* < 0.001). The uncertainty of the sporeling emergence process (U) was higher in mature than immature spores, but both had similar values considering the respective U-range (from 0 to 5.78 and 3.36, respectively; *t*-test: *p* < 0.05) indicating frequencies with several peaks and then emergence processes not concentrated in time.

### 2.3. Transplants

**Figure 5:**
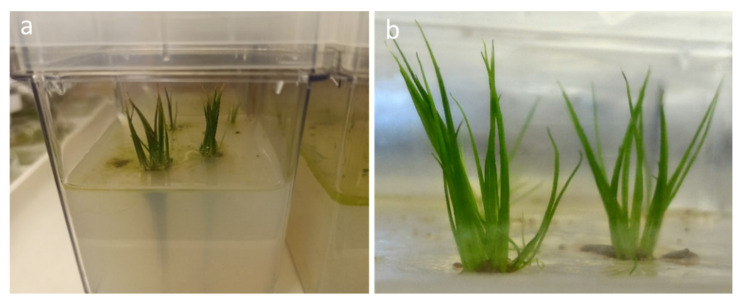
Sporelings of *Isoëtes sabatina* transplanted in plant culture containers Magenta^TM^ GA7 on water-agar (1%) medium (4 cm high) with (**a**) visible roots and (**b**) abundant development of new leaves after 1-year culture.

None of the sporelings transplanted to both lake sediment and quartzite sand have rooted, due mainly to their high tendency to float and then to the difficulty of anchoring them to the substrate, and none of them survived. Instead, sporelings transplanted in water-agar medium showed good results (100%) in term of anchorage to the substrate, rooting (ca. 3–4 cm length), development of new leaves (ca. 4–5 cm length) and high survival (Figure 5).

## 3. Discussion

### 3.1. In Vitro Sporeling Production

This study presents the first successful in vitro propagation protocol for *I. sabatina*, which was critical to establish an ex situ population. To our knowledge, this is the first report of in vitro reproduction of a Mediterranean quillwort. The tested culture media (distilled water and water-agar) were highly effective in terms of percentages of sporeling production, with no significant differences between the two substrates, except for the percentage of sporelings per day (MDE, Table 2). However, by analyzing the propagation process, from sowing to transplanting, the water-agar culture was certainly more effective. In fact, in distilled water cultures, the lack of a solid substrate for the sporelings in which to root and grow required an early transplant. On the contrary, in water-agar cultures, the sporelings were able to root and grow for months before being transplanted. 

A total of 351 sporelings were obtained from the ten adult specimens collected, which were ready for transplanting within about four months after spore sowing. The percentage of sporeling production from mature spores (70%) was higher than the percentages reported for other *Isoëtes* species: 12.3% for *I. coreana* Y.H.Chung and H.K.Choi [14], 15.1% for *I. echinospora* Durieu [33], 57.9% for *I. lacustris* [32], and 63% for *I. cangae* [18].

Mature megaspores of *I. sabatina* germinated with a high percentage (85%) at 20 °C without any pre-treatment, contrarily to other species, such as, for example, *I. echinospora*, which requires 3 months of cold stratification to break megaspore dormancy and germinate [33,34]. This likely reflects the Mediterranean character of *I. sabatina*, showing spore dispersal in summer and sporeling emergence in late summer/early autumn (personal observation), while *I. echinospora* growths in cold lakes at northern latitudes with spore release from October and sporeling development in July [33], requiring a long cold period to germinate. Moreover, this ecological difference reinforces the taxonomic autonomy of *I. sabatina*, which proves to be different from the most morphologically similar species, *I. echinospora*.

Several authors accounted for the developmental stage of the megaspores as a critical factor affecting germination in *I. coreana* [14], *I. malinverniana* [10] and, especially, *I. cangae* with no germination of immature megaspores [18]. However, germination results for *I. sabatina* are only partially in accordance with these results, as no germination was observed exclusively in cultures of spores from beached plants, collected more than one month before maturity, being too small and underdeveloped to germinate. On the contrary, immature megaspores from mature plants germinated with a high percentage (65.6%), even if significantly lower than mature spores (Table 1). 

On the other hand, the maturity stage of the megaspores strongly affected sporeling development. Significant differences were detected in eight out of ten indices used to compare the sporeling emergence from both mature and immature megaspores. In particular, lower percentages of sporeling emergence were recorded for immature spores (Table 2, Figure 4), despite the high germination percentage. However, this may be a secondary effect of the spore developmental stage. Sowing immature megaspores together with mature microspores can lead to the desynchronizing of the spore germination [33]. In this study, microspores germinated soon after sowing, while immature megaspores started to germinate after an average of 77 days, resulting in the low fertilization and poor sporeling development recorded (SE_G_ = 16.7%, Table 2). Contrarily, the synchronized germination of mature megaspores and microspores, with overlapping germination periods, was reflected in successful fertilization and sporeling development (SE_G_ = 85%, Table 2).

These results highlight the longevity of non-fertilized megagametophytes in ex situ cultures (2 years and 10 months). It is known that new archegonia are produced continually throughout the life of the gametophyte until fertilization and, in case this is delayed, the archegonia continue developing till the resources in the storage tissue are exhausted [35]. The number of archegonia observed on a single megagametophyte of *I. sabatina* (48–58, Figure 2d) is much higher than the numbers reported for other species: 12 for *I. lithophila* N.E.Pfeiffer [35], 23 for *I. andicola* (Amstutz) L.D.Gómez [36] and 20–30 for *I. lacustris* [37]. This confirms that the number of archegonia that may develop in a single gametophyte, when fertilization is prevented, is not definite and depends solely upon the abundance of storage tissue in the megaspore [35]. 

Transplants on sand and sediments failed, probably due to the small size of the sporelings (ca. 1 cm) and their tendency to float. They were successful exclusively when sporelings were grown over a water-agar medium, where they showed an evergreen pattern for more than 2 years of ex situ cultivation, developing new green leaves and maintaining the oldest green leaves strongly attached to the plant corm, as reported also for *I. cangae* [18].

### 3.2. The Conservation Challenges for I. sabatina

Current habitat degradation with associated extinction rates show that freshwater ecosystems already face greater pressures than any other ecosystem, and threats will intensify in the future as the exploitation of freshwater resources grows to meet human demand [38]. Therefore, freshwater ecosystems should be of primary concern in the environmental policy-agenda and freshwater biodiversity should be considered as one of the global conservation priorities. In this regard, there is an urgent need for more effective restoration approaches for aquatic plant communities.

*Isoëtes sabatina* is an aquatic quillwort point-endemic to Lake Bracciano (Italy), having a set of characteristics that can be found in most endemic species, which make them vulnerable to anthropogenic threats and/or natural changes [39], e.g., restricted distribution, one small-sized declining population, specific habitat requirements, and necessity of stable and constant environment. These intrinsic characteristics, together with the drastic decline of its habitat since 2017, caused by the 2 m reduction in the lake’s water level, are responsible for this species being increasingly threatened with extinction. It has been suggested that below a certain threshold size, local populations enter an extinction vortex, i.e., a spiral of ever-decreasing population size [40,41]. A 60% drop in the population had already been recorded for *I. sabatina* but its minimum threshold size is unknown. Hence, it is of critical importance to plan and implement an integrated strategy involving both ex situ and in situ conservation efforts aimed at improving the status of its habitat and wild population to save this endemic quillwort from the brink of extinction [16]. 

The development of an effective in vitro propagation protocol and the production of an adequate number of plants was the first challenge we have faced for the conservation of *I. sabatina*, the first mandatory step within a larger reintroduction or reinforcement program [28,42]. The importance of in vitro tools to complement other ex situ/in situ methods to save plants from extinction is relevant and particularly useful in the case of rare and endangered species [42,43]. On the other hand, there is an urgent need to proceed with ex situ spore conservation, to provide a long-term (extinction-proof) safety backup for the species and its genetic diversity, and, especially, with effective in situ conservation actions urgently required to avoid further population decline and to definitively halt the risk of its extinction.

Based on this, the main conservation challenge for *I. sabatina* is to plan and carry out an in situ conservation program, including habitat restoration and reintroduction or reinforcement activities, as recently reported for *I. malinverniana* [10], to increase the size of the depleted wild population using innovative solutions able to reduce the negative effects induced by future climatic uncertainty and water level variations. 

## 4. Materials and Methods

### 4.1. Study Area 

Lake Bracciano is one of the 12 volcanic lakes occurring in south-central Italy, located in the northern part of the Latium Region (central Italy) at an altitude of 164 m a.s.l. It is one of the major lakes of Italy, having a perimeter of approximately 32 km and a surface area of 56.76 km^2^ with a maximum depth of 165 m. Its inflow is from precipitation runoff and percolation, as well as underground springs. It is a deep meso-oligotrophic hard water lake, like other Italian volcanic lakes [44,45], with a circular shape related to its volcanic origins.

### 4.2. Plant Material

Plants of *I. sabatina* were collected on 28 June 2017 from the species’ only known population at the south-eastern coast of Lake Bracciano (Lungolago di Polline, voucher specimen in the Herbarium RO). To avoid further population depletion, only 10 adult specimens were collected, selected among those closest to the coastline that would have emerged and therefore died due to the further summer drop in water level, together with 12 beached plants. The plants were carefully removed from the bottom of the lake together with large root balls to preserve the integrity of their root system. 

These plants were taken immediately to the Tuscia Germplasm Bank where they were grown until maturity. Lake sediment upon a gravel substrate was used as a growing medium and the plants were placed in an aquarium inside a cooled incubator (Panasonic MIR-154-PE, Osaka, Japan), at alternate temperatures (26/20 °C) under a 16/8 h photoperiod.

### 4.3. Spore Collection Ex Situ

*Isoëtes sabatina* is a heterosporous lycophyte, hence it produces both microspores and megaspores in separate sporangia, in different leaves, i.e., micro- and mega-sporophylls, respectively. Each sporophyll bears a single flattened sporangium incompletely covered by a velum within a cavity (fovea) between the ligule and leaf base on their adaxial surface. The sporophylls are arranged spirally on the corm, with microsporangia mainly in the internal ones. 

Sporangia were removed from the beached plants the day after the collection as they were too damaged to be cultivated. A total of 20 megasporangia and 10 microsporangia were collected, containing only small immature spores; the megaspores (ca. 300) were underdeveloped and with a thin siliceous layer, without ornamentation. 

To collect mature sporangia, the other plants were grown for more than a month until the prevalence of erect patent sporophylls was observed, as this was considered an indicator of the occurrence of mature larger megaspores. The sporophylls were manually detached from the plants and sporangia were gently removed from the leaf base under a stereomicroscope (Nikon SMZ1000, Tokyo, Japan) using fine tweezers, taking great care not to break the sporangial wall. All intact microsporangia and megasporangia (12 and 35, respectively), and all the megaspores from ruptured sporangia were collected from each plant. All of the sporangia were mixed to maximize the genetic variability of reproduced sporelings.

### 4.4. In vitro Mixed Culture of Mega- and Microspores

All the intact sporangia collected from both beached and cultivated plants were superficially sterilized to obtain naturally sterile spores avoiding the need for extremely difficult sterilization of the microspores for in vitro culture. Sporangia were washed and soaked in water for 1 h under a laminar flow hood, then sterilized for 10 min in a solution of Sodium Hypochlorite (NaClO) with 5% of available chlorine and rinsed three times with sterile distilled water. After the final rinse, sporangia were soaked again in sterile distilled water to avoid dehydration. The mature megaspores collected from sporangia ruptured during the spore collection were sterilized for 5 min according to the protocol described above.

Intact mega- and microsporangia were opened directly in Petri dishes (6 cm diameter) to avoid spore loss. The sporangial wall was ruptured by a scalpel and sterile distilled water was added for spore release, mixing, and allowing fertilization.

Immature and undeveloped micro- and megasporangia from beached plants were mixed and sown in plates with water-agar medium. 

Two different substrates were tested for the germination of mature megaspores and sporeling emergence:

(1) Water-agar (1%) medium, as described by [31];
(2) Sterile distilled water, as described by [14,46].

A total of 328 mature megaspores collected from mature (brown) megasporangia from cultivated plants were incubated in Petri dishes with water-agar (1%) medium, in a mixed culture with a pool of microspores from intact (brown) mature sporangia. All of the megaspores (104) collected from ruptured sporangia were incubated in Petri dishes with distilled water, in a mixed culture with a pool of microspores from intact mature sporangia. 

To evaluate the influence of the megaspore developmental stage on germination and sporeling development, all of the well-developed immature (white) megaspores (205) collected from intact immature (pale brown) megasporangia from cultivated plants were split in different plates with water-agar (1%) medium and mixed with mature microspores from intact mature sporangia. 

All of the plates were closed with Parafilm to prevent water loss. Sterile distilled water (ca. 1.5 mL per plate) was added monthly to allow fertilization in water-agar cultures. The experiments were conducted under the same culture conditions inside a cooled incubator (Panasonic MIR-154-PE, Osaka, Japan) at a constant temperature (20 °C) and 12/12 h photoperiod according to [10]. Different temperatures were not tested in this study because of the reduced spore availability and the urgent need to produce plants to reinforce the remnant small population.

### 4.5. Evaluation of Spore Germination and Sporeling Development

Spore germination and sporeling appearance were scored daily using a 1 × 1 cm grid under a stereoscopic microscope (Nikon SMZ1000, Tokyo, Japan). Both megaspore germination and sporeling emergence were recorded separately for mature and immature megaspores. Megaspore germination percentages 120 days after sowing (total germination, G) and the number of days to first germination (T_G_) were recorded. 

The mega- and microspores of *I. sabatina* are trilete, 545–645 μm and 29–33 μm in diameter, respectively (Figure 2a) [9]. They both germinate endosporically to produce female and male gametophytes, respectively. Megaspores were considered to have germinated when the wall breaks open along the trilete aperture to expose the apical gametophytic tissue (Figure 2b). Thirty megaspores were photographed immediately after sowing and after germination, under a Nikon SMZ 1000 stereomicroscope with a digital camera (Nikon DXM1200, Tokyo, Japan). Megaspore equatorial diameter was measured to calculate diameter increase due to germination, using the software LUCIA Measurement version 4.80 (Laboratory Imaging Ltd., Praha, Czech Republic) for NIKON Instruments. The number of archegonia developed on non-fertilized megagametophytes was recorded. Microspores were considered germinated when there were either four cells with spermatozoids inside a microspore, or when they were empty (Figure 2c). All of the sown megaspores were considered to quantify germination, while the percentage of microspores germinating was determined from five random fields of view per Petri dish (~200 spores per field) [32]. 

After fertilization, the development of the embryo begins. The embryogeny in *Isoëtes* is endoscopic, as in other lycophytes (*Lycopodium* and *Selaginella)*, *Marattiaceae* and some *Ophioglossaceae* [35,47]: the embryo develops from the inner of the two cells resulting from the first division of the zygote, with the foot remaining within the spore wall and the shoot and the root apices located near the archegonium neck [47]. The embryo grows rapidly with the first leaf of the sporeling bursting out of the megagametophyte by the seventh day [48]. Sporeling emergence was scored daily for 3 months, considering the time in which the first leaf starts protruding from the megagametophytic tissue (Figure 4a,b). The emergence of the first roots was scored in ten sporelings and their length was measured as described above for megaspores.

### 4.6. Calculation of Sporeling Emergence Indices

For the evaluation of the sporeling emergence process and to compare data for different substrates and megaspore developmental stages, the following indices were calculated according to formulas in [49,50], modified as follows:

- *Sporeling emergence percentage* (SE, in percent), calculated on the number of sown megaspores, and the number of germinated megaspores (SE_G_, in percent);
- *Time for first sporeling emergence* (T_1,_ in days);
- *Mean daily emergence* (MDE, in percent), calculated by the expression: MDE=SETn, where *T_n_* is the total number of days required to reach the final sporeling emergence percentage (*SE*);
- *Mean emergence time* (MET, in days) indicates the average length of time required for maximum sporelings emergence and was calculated according to the following formula: $MET=\frac{\sum_{i=1}^{k}n_{i}t_{i}}{\sum_{i=1}^{k}n_{i}}$, where *n_i_* is the number of sporelings newly emerged at time *t_i_*;
- *Mean emergence rate* (MER, in day^−1^) is the average number of sporelings emerged per day, calculated as the reciprocal of MET;
- *Coefficient of variation of emergence time* (CV_t_), calculated by the expression CVt=SDtMET×100, where *SDt* is the standard deviation of the emergence time and *MET* is the mean emergence time;
- *Emergence Index* (EI, number of sporelings per day), calculated by the expression EI=∑i=1kniti, where *n_i_* is the number of sporelings emerged at time *t_i_*;
- *Peak value *(PV, sporelings/day) is the accumulated number of sporelings at the point on the emergence curve at which the rate of emergence starts to decrease. It is calculated as the maximum quotient obtained by dividing successive cumulative emergence values by the respective incubation time;
- *Uncertainty of the sporeling emergence process* (U, in bit), i.e., the uncertainty associated with the distribution of the relative frequency of emergence (*fi*), calculated through the formula: $U=-\overset{k}{\underset{i=1}{\sum }}fi\log_{2}fi$, being $fi=\frac{n_{i}}{\sum_{i=1}^{k}n_{i}}$, where *n_i_* is the number of sporelings emerged at time *t_i_*. It can assume values spanning from 0 and log_2_*n*, with *n* being the total number of sporelings emerged.

In distilled water cultures, only SE, SE_G_, T_1_ and MDE were calculated because of the difficulty of recording the daily emergence of new sporelings due to their floating and movement on the water. 

The length of the first two fully developed leaves was measured in ten sporelings. The *relative growth rate* (RGR, in mm/day) was calculated using the following equation: $RGR=\frac{\left(\ln L_{2}-\ln L_{1}\right)}{t_{2}-t_{1}}$, where *L*_1_ and *L*_2_ are leaf lengths (in mm) at times *t*_1_ and *t*_2_ (in days), respectively [51]. 

### 4.7. Transplants

Three different substrates were tested for further growth:

(1) Lake sediment was homogeneously distributed at the bottom of glass containers (20 × 10 × 10 cm), covered with a thin layer of sterilized quartzite sand to avoid the spread of debris according to [18], and then covered with distilled water up to a height of ca. 3 cm;
(2) Quartzite sand was homogeneously distributed at the bottom of the glass container and covered with distilled water up to a height of ca. 3 cm;
(3) Water-agar (1%) medium (4 cm high) in plant culture containers Magenta^TM^ GA7 (7.6 × 7.6 × 10.2 cm), wetted with only 5 mL of distilled water.

Twenty sporelings, with at least two leaves with an approximate length of 1 cm and a root of at least 0.5 cm length, were used for each substrate. The transplanted sporelings were kept in the cooled incubator at a constant temperature (22 °C) and 12/12 h photoperiod. Sporeling anchorage to the substrate, rooting and survival were recorded weekly.

### 4.8. Statistical Analysis

Megaspore germination and sporeling development data for different substrates and megaspore developmental stages were analyzed using one-way ANOVA (after a check of Bartlett’s statistics) using GraphPad Prism version 5.04 (GraphPad Software, San Diego, CA, USA), followed by Tukey’s multiple comparison test to assess the occurrence of statistically significant differences among species. When Bartlett’s statistics revealed a non-homoscedasticity of variance (*p* < 0.05), then the non-parametric Kruskal–Wallis test for independent samples (followed by Dunn’s multiple comparison test) was used. Sporeling emergence indices for mature and immature megaspores were analyzed through a *t*-test. Before analysis, all data were normalized by arcsine transformation, but they are expressed in the tables as non-transformed data. The results are statistically significant, with *p* lower than the significance level of 0.05.

## 5. Conclusions

In this study, we investigated the reproductive biology and developed a method to propagate many *I. sabatina* sporelings and to establish an ex situ population. In vitro culture on water-agar medium is a low-cost method that has proved effective in terms of percentages of sporeling production and their longevity in culture, providing a solid substrate in which the sporelings can root and grow for months before being transplanted. 

As a take-home message from this research, a simple protocol is provided for the successful propagation of *I. sabatina*. The collection of intact sporangia is recommended, so as to avoid the difficult sterilization method for microspores. Mature spores should be sown on water-agar medium in mixed culture. For propagation purposes, we suggest sowing mature spores directly in large in vitro containers filled with a 4–5 cm layer of water-agar (1%) medium, instead of Petri dishes, to avoid the need for sporeling transplanting, which can lead to damage to sporeling roots or fungal contamination. Cultures should be incubated at a temperature of 20 °C under a photoperiod with 12 h of light and 12 h of dark.

To our knowledge, this is the first report of the in vitro reproduction of a Mediterranean quillwort and this method could be useful to also propagate other Mediterranean *Isoëtes*.

This is the first key action for the effective conservation of this critically endangered aquatic quillwort endemic to Italy, providing critical steps towards future measures to recover the small declining population from extinction. The translocation in nature of the specimens produced under the ex situ conservation program would make it possible to reinforce the wild population of *I. sabatina* with an increase in population size of at least 25%. A multi-year program that combines ex situ germination and in situ transplantations will be implemented over the next 5 years, ensuring the survival of the species.

## Figures and Tables

**Table 1:** Germination percentage (G) and time for the first germination (T_G_) of megaspores of *Isoëtes sabatina* in distilled water and water-agar cultures. All values are reported as mean ± SD; values followed by the same letter are not significantly different at the 5% level of probability, as determined by Dunn’s test. Significance determined by the Kruskal–Wallis test is reported in the last column as follows, **: 0.001 < *p* < 0.01; *: 0.01 < *p* < 0.05.

Substrates	Distilled Water	Water-Agar Medium		
Megaspore Developmental Stage	Mature (*n* = 104)	Mature (*n* = 361)	Immature (*n* = 205)	
G (%)	80.1 ± 5.0 a	82.1 ± 13.1 a	65.6 ± 16.1 b	*
T_G_ (days)	41.7 ± 1.5 a	48.3 ± 9.3 a	77.0 ± 6.7 b	**

**Table 2:** Indices for the evaluation of sporeling emergence from mature and immature megaspores of *Isoëtes sabatina* in water-agar cultures. SE: sporeling emergence percentage, calculated for the total number of sown megaspores; SE_G_: sporeling emergence percentage, calculated on the total number of the germinated megaspores; T_1_: time for first sporeling emergence; MDE: mean daily emergence; MET: mean emergence time; MER: mean emergence rate; CVt: Coefficient of variation of emergence time; EI: emergence index; PV: peak value; U: uncertainty of the sporeling emergence process. All values are reported as mean ± SD; values followed by the same letter are not significantly different at the 5% level of probability, as determined by Dunn’s test. Significance determined by 1-way ANOVA or Kruskal–Wallis test (SE, SE_G_, T_1_, MDE) and *t*-test (MET, MER, CVt, EI, PV, U) are reported in the last column as follows: ***: *p* < 0.001; **: 0.001 < *p* < 0.01; *: 0.01 < *p* < 0.05; ns: *p* > 0.05.

Sporeling Emergence Indices	Distilled Water	Water-Agar Medium		
	Mature Megaspores	Mature Megaspores	Immature Megaspores	
**SE** (%)	64.5 ± 8.3a	69.6 ± 15.5a	11.6 ± 7.9b	**
**SE_G_** (%)	80.4 ± 5.4a	85.0 ± 12.7a	16.7 ± 7.9b	**
**T_1_** (day)	52.7 ± 3.1a	59.5 ± 8.5a	85.0 ± 6.7b	*
**MDE** (% sporelings/day)	0.8 ± 0.2a	0.6 ± 0.1b	0.1 ± 0.1c	***
**MET** (day)	-	82.9 ± 6.7	87.9 ± 5.0	*ns*
**MER** (day^−1^)	-	0.012 ± 0.001	0.011 ± 0.001	*ns*
**CVt** (%)	-	12.1 ± 1.8	6.9 ± 4.3	*
**EI** (n. sporelings/day)	-	0.8 ± 0.4	0.1 ± 0.1	*
**PV** (day^−1^)	-	0.70 ± 0.11	0.12 ± 0.07	***
**U** (bit)	-	3.1 ± 0.3	1.7 ± 1.1	*

## References

[1] Troia A., Pereira J.B., Kim C., Taylor W.C. (2016). The genus *Isoëtes* (Isoetaceae): A provisional checklist of the accepted and unresolved taxa. Phytotaxa.

[2] Brunton D.F., Troia A. (2018). Global review of recent taxonomic research into *Isoëtes* (Isoetaceae), with implications for biogeography and conservation. Fern Gaz..

[3] Hoot S.B., Taylor W.C., Napier N.S. (2006). Phylogeny and biogeography of *Isoëtes* (Isoëtaceae) based on nuclear and chloroplast DNA sequence data. Syst. Bot..

[4] Seddon B. (1965). Occurrence of *Isoëtes echinospora* in eutrophic lakes in Wales. Ecology.

[5] Hickey R.J., Macluf C., Taylor W.C. (2003). A re-evaluation of *Isoëtes savatieri* Franchet in Argentina and Chile. Am. Fern J..

[6] Liu X., Wang J.-Y., Wang Q.-F. (2005). Current status and conservation strategies for *Isoëtes* in China: A case study for the conservation of threatened aquatic plants. Oryx.

[7] Kim C., Na H.R., Choi H.-K. (2008). Genetic diversity and population structure of endangered *Isoëtes coreana* in South Korea based on RAPD analysis. Aquat. Bot..

[8] Pereira J.B.S., Salino A., Arruda A., Stutzel T. (2016). Two New species of *Isoëtes* (Isoetaceae) from northern Brazil. Phytotaxa.

[9] Troia A., Azzella M.M. (2013). *Isoëtes sabatina* (Isoëtaceae, Lycopodiophyta), a new aquatic species from central Italy. Plant Biosyst..

[10] Abeli T., Cauzzi P., Rossi G., Pistoja F., Mucciarelli M. (2018). A gleam of hope for the critically endangered *Isoëtes malinverniana*: Use of small scale translocations to guide conservation planning. Aquat. Conserv..

[11] Jacobsen D., Dangles O. (2017). Ecology of High Altitude Waters.

[12] Free G., Bowman J., McGarrigle M., Caroni R., Donnelly K., Tierney D., Trodd W., Little R. (2009). The identification, characterization and conservation value of isoetid lakes in Ireland. Aquat. Conserv..

[13] García Criado M., Väre H., Nieto A., Bento Elias R., Dyer R., Ivanenko Y., Ivanova D., Lansdown R., Molina J.A., Rouhan G. (2017). European Red List of Lycopods and Ferns.

[14] Oh M.J., Kim C., Na H.R., Shin H., Liu J.R., Choi H.-K., Kim S.W. (2013). High frequency sporophytes regeneration from the spore culture of the endangered aquatic fern *Isoëtes coreana*. Am. J. Plant Sci..

[15] Christenhusz M., Bento Elias R., Dyer R., Ivanenko Y., Rouhan G., Rumsey F., Väre H. (2017). Isoëtes sabatina. The IUCN Red List of Threatened Species 2017.

[16] Orsenigo S., Montagnani C., Fenu G., Gargano D., Peruzzi L., Abeli T., Alessandrini A., Bacchetta G., Bartolucci F., Bovio M. (2018). Red Listing plants under full national responsibility: Extinction risks and threats in the vascular flora endemic to Italy. Biol. Conserv..

[17] Barni E., Minuzzo C., Gatto F., Lonati M., Abeli T., Amosso C., Rossi G., Siniscalco C. (2013). Estimating influence of environmental quality and management of channels on survival of a threatened endemic quillwort. Aquat. Bot..

[18] Caldeira C.F., Abranches C.B., Gasutauer M., Ramos S., Guimarães J.T.F., Pereira J.B.S., Siqueira J.O. (2019). Sporeling regeneration and ex situ growth of *Isoëtes cangae* (Isoetaceae): Initial steps towards the conservation of a rare Amazonian quillwort. Aquat. Bot..

[19] Giuffrida A., Taylor M. (2017). Romans threatened with water rationing as Italy’s heatwave drags on. The Guardian.

[20] Horowitz J. (2017). Rome, City of Ancient Aqueducts, Faces Water Rationing. The New York Times.

[21] Baccetti N., Bellucci V., Bernabei S., Bianco P., Braca G., Bussettini M., Cascone C., Ciccarese L., D’Antoni S., Grignetti A. (2017). Analisi e Valutazione Dello Stato Ambientale del Lago di Bracciano Riferito All’estate 2017.

[22] Hoerling M., Eischeid J., Perlwitz J., Quan X., Zhang T., Pegion P. (2012). On the increased frequency of Mediterranean drought. J. Clim..

[23] Planton S., Lionello P., Artale V., Aznar R., Carrillo A., Colin J., Congedi L., Dubois C., Elizalde A., Gualdi S., Lionello P. (2012). Chapter 8. The Climate of the Mediterranean Region in Future Climate Projections. The Climate of the Mediterranean Region.

[24] Magrini S., Olmati C., Onofri S., Scoppola A. (2010). Recovery of viable germplasm from herbarium specimens of *Osmunda regalis* L.. Am. Fern J..

[25] Magrini S. (2011). Herbaria as useful spore banks for integrated conservation strategies of pteridophytic diversity. Plant Biosyst..

[26] Magrini S., Scoppola A. (2012). Agravitropic growth of the early leaves of apogamous sporophytes of *Dryopteris tyrrhena*. Am. Fern J..

[27] Magrini S., Scoppola A. (2012). First results from conservation studies of chlorophyllous spores of the Royal fern (*Osmunda regalis*, Osmundaceae). Cryobiology.

[28] Fay M.F. (1994). In what situations is in vitro culture appropriate to plant conservations?. Biodivers. Conserv..

[29] Sarasan V., Cripps R., Ramsay M.M., Atherton C., McMichen M., Prendergast G., Rowntree J.K. (2006). Conservation in vitro of threatened plants—Progress in the past decade. In Vitro Cell. Dev. Biol. Plant.

[30] Engelmann F. (2011). Use of biotechnologies for the conservation of plant biodiversity. In Vitro Cell. Dev. Biol. Plant.

[31] Abeli T., Mucciarelli M. (2010). Notes on the Natural History and reproductive biology of *Isoëtes malinverniana*. Am. Fern J..

[32] Čtvrtlíková M., Znachor P., Vrba J. (2014). The effect of temperature on the phenology of germination of *Isoëtes lacustris*. Preslia.

[33] Čtvrtlíková M., Znachor P., Nedoma J., Vrba J. (2012). The effect of temperature on the phenology of germination of *Isoëtes echinospora*. Preslia.

[34] Kott L.S., Britton D.M. (1982). A comparative study of spore germination of some *Isoëtes* species of northeastern North America. Can. J. Bot..

[35] La Motte C. (1933). Morphology of the Megagametophyte and the Embryo Sporophyte of *Isoetes lithophila*. Am. J. Bot..

[36] Karrfalt E. (1999). Some Observations on the Reproductive Anatomy of *Isoetes andicola*. Am. Fern J..

[37] Kienitz-Gerloff F. (1881). Über Wachstum und Zelltheilung in der Entwickelung des Embryos von *Isoetes lacustris*. Bot. Zeit..

[38] Reid A.J., Carlson A.K., Creed I.F., Eliason E.J., Gell P.A., Johnson P.T.J., Kidd K.A., MacCormack T.J., Olden J.D., Ormerod S.J. (2019). Emerging threats and persistent conservation challenges for freshwater biodiversity. Biol. Rev..

[39] Işik K. (2011). Rare and endemic species: Why are they prone to extinction?. Turk. J. Bot..

[40] Gilpin M.E., Soulé M.E., Soulé M.E. (1986). Minimum viable populations: The processes of species extinctions. Conservation Biology: The Science of Scarcity and Diversity.

[41] Lamont B.B., Klinkhamer P.G.L., Witkowski E.T.F. (1993). Population fragmentation may reduce fertility to zero in *Banksia goodii*—A demonstration of the Allee effect. Oecologia.

[42] Coelho N., Gonçalves S., Romano A. (2020). Endemic plant species conservation: Biotechnological approaches. Plants.

[43] Barnicoat H., Cripps R., Kendon J., Sarasan V. (2011). Conservation in vitro of rare and threatened ferns—Case studies of biodiversity hotspot and island species. In Vitro Cell. Dev. Biol. Plant.

[44] Bolpagni R., Laini A., Azzella M.M. (2016). Short-term dynamics of submerged aquatic vegetation diversity and abundance in deep lakes. Appl. Veg. Sci..

[45] Azzella M.M., Bresciani M., Nizzoli N., Bolpagni R. (2017). Aquatic vegetation in deep lakes: Macrophyte co-occurrence patterns and environmental determinants. J. Limnol..

[46] Taylor W.C., Luebke N.T. (1986). Germinating spores and growing sporelings of aquatic *Isoëtes*. Am. Fern J..

[47] Niklas K.J., Leck M.A., Parker V.T., Simpson R.L. (2008). Embryo morphology and seedling evolution. Seedling Ecology and Evolution.

[48] Wardlaw C.W., Addicott F.T., Lang A., Ruhland W. (1965). Physiology of embryonic development in cormophytes. Encyclopedia of Plant Physiology XV Part 1. Differentiation and Development.

[49] Ranal M.A., Santana D.G. (2006). How and why to measure the germination process?. Rev. Bras. Bot..

[50] Ranal M.A., Garcia De Santana D., Resende Ferreira W., Mendes-Rodrigues C. (2009). Calculating germination measurements and organizing spreadsheets. Rev. Bras. Bot..

[51] Hoffmann W.A., Poorter H. (2002). Avoiding bias in calculations of Relative Growth Rate. Ann. Bot..

